# Dissecting the Medical Student Approach to Chest Pain: A Cross-Sectional Study Focusing on Aortic Dissection

**DOI:** 10.7759/cureus.29050

**Published:** 2022-09-11

**Authors:** Alex Teasdale

**Affiliations:** 1 General Surgery, Glangwilli General Hospital, Swansea, GBR

**Keywords:** symptom assessment, cardio thoracic surgery, student education, type a aortic dissection, cardiac chest pain

## Abstract

Background and purpose: To understand if medical students consider rarer, but highly fatal conditions, such as acute aortic dissection, when assessing chest pain.

Methods:An online virtual clinical case (in two 'stages') was distributed to medical students. Stage one presented a chest pain scenario, asking participants to give relevant questions, clinical findings, investigations, and differential diagnoses. In the second stage, students were given a detailed case, populated with the aortic dissection ‘red flags’ and asked to re-evaluate their differential diagnosis and investigations. A total of 113 students in their final two years of study, across six United Kingdom (UK) medical schools, were accepted into the study.

Results: During stage one, students were able to assess chest pain with radiation (93%), character (83%), and onset (89%), which were asked commonly. However, students failed to enquire on severity (38%) and important risk factors such as connective tissue disorders (4%) and hypertension (46%), or clinical signs such as blood pressure differences (5%). Myocardial infarction (97%) and pulmonary embolism (78%) were the most considered differentials with acute aortic dissection only considered by 31%. However, in stage two, 73% recognised the acute aortic dissection and 76% were able to request a CT thorax.

Conclusions: Students effectively consider myocardial infarction and pulmonary embolism when assessing chest pain, but often fail to examine acute aortic dissection. This could lead to delayed treatment of a cataclysmic event and may be due to a deficiency in diagnostic synthesis rather than a lack of knowledge. More can be done to ensure these conditions form part of their diagnostic workup.

## Introduction

Chest pain is a common presenting complaint seen in the emergency department and primary care settings, with around 1-2% of adults in the United Kingdom (UK) attending primary care with this problem each year [1]. Unfortunately there are many different causes of chest pain and, despite many years of experience, clinicians can, and do, make errors of diagnosis. These occasionally lead to a delay in treatment or ineffective treatment being offered. In order to reach a likely diagnosis, a clinician will embark on a continuous process of gathering information from the history, examination, and investigations performed. A clinician will consider multiple possible diagnosis when information is limited, but this list will be refined as the likelihood of one diagnosis over another changes, as more information becomes available. One of the most significant reasons to make an error in diagnosis is because of a propensity to stop looking for an alternative diagnosis when a more obvious first one is reached [2]. One such condition which might suffer from this folly is acute aortic dissection, which, although relatively uncommon, is a major killer.

Approximately 40% of patients who suffer an acute aortic dissection die within seconds, but the mortality for those who survive the initial period increases by 1% every hour [3]. A dissection most commonly presents acutely as a cataclysmic event in the emergency department, but can present more subtly and is often missed, or the diagnosis delayed. The disease begins as a spontaneous rip in the adventitia or intima of the wall of the aorta. Pulsatile blood rapidly flows in, dissecting the layers within the aortic wall, resulting in the creation of a false lumen alongside the true lumen, or a double-barrel aorta. As the aorta is now torn, it is immensely weakened and prone to rupture, a scenario which is most usually fatal [4].

There are two types of classification for aortic dissection, Stanford and DeBakey, and both are based on the anatomical positioning of the dissection. The most commonly used is Stanford, in which type A is classified as those which occur in the ascending aorta, and type B being the rest [5]. Both of these can be classified further depending on whether or not they are acute (< 2 weeks) or chronic (>2 weeks) [6]. Although there is no etiological classification system, it is important to be aware of the fact that a dissection can be spontaneous, genetic (Marfans or Ehlers-Danlos syndrome), or traumatic. Gold standard imaging of a type A dissection is a CT thorax, although some dissections could be noted on an echocardiogram and in some cases, widening of the mediastinum may be seen on a plain chest radiograph [7].

Successful treatment of both classifications requires a rapid diagnosis, and often relies on surgical repair or aggressive medical management, as well as monitoring for the remainder of the patient's life [8]. To increase the chance of early diagnosis, it is vital that important risk factors and 'red flags' are elucidated and relevant investigations are undertaken. According to figures published by the International Registry of Acute Aortic Dissection (IRAD), patients are more commonly males with an average age of 63 [9]. The most common risk factors are hypertension (72%), atherosclerosis (31%), and Marfans syndrome (5%). Patients usually complain of a sudden onset, sharp and severe chest pain, occasionally with radiation to the back. Pulse deficits are seen in 30% with type A dissection [7].

It is vitally important that the presentation of acute aortic dissection is not missed, and that clinicians are properly trained to identify it quickly and effectively. Although teaching methods and philosophies are constantly changing and evolving, especially in medicine, the big question remains: what is the most effective way to teach and achieve the best results from the students? We have hypothesized that medical students are being trained to consider common presentations, rather than ruling out rarer, but more deadly conditions. To test this, we produced an online virtual clinical case that aimed to better understand how medical students, who are about to become junior doctors, assess chest pain and if they are able to consider acute aortic dissection as a diagnosis in the face of more common, but less fatal alternatives.

## Materials and methods

Using a cross-sectional study design, an online virtual clinical case consisting of two ‘stages’ was created using SurveyMonkey (Momentive Inc., San Mateo, California, United States). This platform was chosen due to its simplicity and because users did not need to create an account to complete it, thus increasing uptake. The case was designed to follow a linear format, where students could not enter the second stage without first completing the first. They were also unable to return to a previous page, which minimised the risk of them receiving helpful information prematurely, reducing bias.

The virtual case was distributed to medical students in their final two years of study across six UK medical schools: Cardiff, Exeter, Leeds, Manchester, Swansea, and East Anglia. Dissemination of the survey was achieved by contacting the local undergraduate leads for each respective medical school and receiving an email list of students in the final two years of study. Level of study was self-reported by the students, who were asked to provide this information at the start of the survey. The spread of universities allowed for a mixture of graduate, undergraduate, problem-based learning, and case-based learning courses. Students were recruited anonymously and voluntarily and ethical principles as per the World Medical Association Declaration of Helsinki were adhered to [10]. Stage one of the study asked participants to work through a basic, but realistic, scenario; ‘A 42-year-old lady is brought to the emergency department by ambulance with chest pain’. From this, they were asked, using free text boxes to avoid bias, how they might clerk the patient by giving relevant questions, clinical findings, and investigations. This would lead them to suggest the differential diagnoses they felt were most important to consider for this patient.

The students would then enter the second stage of the study. Here they were given a much more detailed case, presented in a similar format to medical school exams and populated with the aortic dissection ‘red flags’, presented in a linear fashion, and based on the American College of Cardiology (ACC) 2010 guidelines [11]. The case read:

‘A 42-year-old lady is brought to the emergency department by ambulance with chest pain. This 42-year-old lady was previously well apart from hypertension during her last pregnancy. Within 15 minutes of breast feeding her child, she developed sudden onset severe (rated 10/10 on a verbal numeric rating scale) chest pain and collapsed in the bathroom. The pain was found to radiate to her back. Her family history is unremarkable apart from her grandmother who passed away suddenly at the age of 55 with an assumed heart attack. She has a 10-pack year smoking history and is not diabetic. On examination she is now pain free, but is cold and clammy. Her left arm blood pressure is 100/60 mmHg and her right is 80/60 mmHg. Her heart rate is 110 beats per minute and saturations are 98% on room air. She has a murmur and her chest was clear on auscultation. Her ECG showed a sinus tachycardia but was otherwise normal. Her chest X-ray was unremarkable'

With this new information, they were asked if they wished to re-evaluate their investigations and differential diagnoses. The responses were collated and analysed in Microsoft Excel (Microsoft Corporation, Redmond, Washington, United States). 

Exclusion criteria

Students not in their final two years of study, or who failed to complete both stages of the case were excluded from the study to ensure an appropriate level of training and accurate comparison between the two stages.

## Results

Overall, 197 students began the case. A total of 71 students were removed as they were not in their final two years of study and therefore were unlikely to have enough cardiology or cardiothoracic experience. A further 13 were removed as they did not complete both stages of the case. In total, 113 participants were accepted into the study. 

During stage one, 93% asked about radiation and 89% about onset; however, only 38% asked about severity, another red flag of acute aortic dissection. A total of 83% of students discussed the ‘character’ of the pain, for example if it was dull, sharp, or tearing. As few as 5% stated they would look for blood pressure differences between arms or pulse deficits; however, 23% were already considering the importance of a new murmur, yet another potential red flag for acute aortic dissection. A total of 46% of the respondents were able to enquire about a past history of hypertension and 79% about smoking, although only 4% thought about connective tissue disorders, such as Ehlers-Danlos or Marfans syndrome.

**Figure 1:**
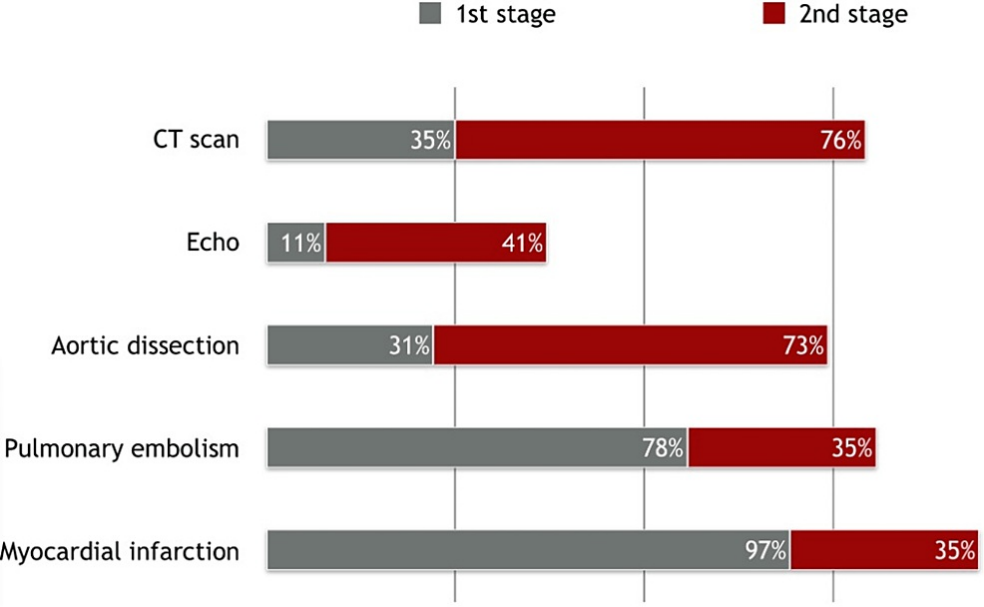
A graphic representation of the key student responses regarding aortic dissection between the first and second stages of the case.

Unsurprisingly, 97% chose an electrocardiogram as their initial investigation of choice with a plain chest radiograph the second most popular, suggested by 78% of the cohort. Importantly for acute aortic dissection, 35% of the cohort suggested a CT scan and 11% an echocardiogram (including Focused Assessment with Sonography in Trauma (FAST) scans). This correlates closely with the 31% of the cohort who included acute aortic dissection as a differential. Many students suggested blood tests, most relevant being troponin-T (92%) and D-dimer (38%). Myocardial infarction and pulmonary embolism were diagnosed by 97% and 78% of the students, respectively, and were the most common differentials, as it may be reasonably expected. However, only 31% gave acute aortic dissection as a possible differential, significantly lower than musculoskeletal pain (63%), gastro-esophageal reflux disease (62%), and pneumonia (52%). After being given the red flags, 76% now chose to request a CT and 73%, more than double the previous section, recognized that the patient was suffering as an acute aortic dissection rather than pulmonary embolism or myocardial infarction (as shown in Figure 1).

## Discussion

Although nearly three-quarters of students were able to identify the dissection when given red flags, the fact that less than a third considered it from the outset serves to highlight that despite its frighteningly high mortality rates, aortic dissection does not get the same level of recognition as other conditions. This could mean that patients with an acute aortic dissection are more likely to be under-investigated, missed and put at high risk. However, the more common conditions, such as myocardial infarction and pulmonary embolism, which receive a high level of focus at medical school in the UK, would appear to be well recognized by the students. Over three-quarters of the students suggested myocardial infarction or pulmonary embolism as the main differential for chest pain and most students were able to ask appropriate questions regarding chest pain, with the majority resorting to the popular mnemonic, SOCRATES (Site, onset, character, radiation, associated symptoms, time course, exacerbating/relieving factors, and severity). 

The second stage of the study could potentially shed some light on the reasoning for this and imply a deficiency in diagnostic synthesis rather than a lack of knowledge. Given the ‘reg flag’ signs and symptoms, the rate of recognition of acute aortic dissection went up dramatically. This suggests that students are being effectively taught about dissections, or encountering them in their studies, and are able to recognize an obvious case when presented with common signs. However, a number of these signs and symptoms may not be elucidated from the patient early enough if the clinician is not looking for them, for example, a new murmur or a noticeable blood pressure difference between arms. It may be difficult to know if a heart murmur is new, but if it has not previously been documented, a high index of suspicion is warranted. It is of course, unreasonable to suggest that every patient with chest pain should receive a CT scan. However, a more comprehensive understanding of serious risk factors for dissection and checking blood pressure on both arms and is not a dramatic addition to the traditional chest pain clerking, although we appreciate most ED departments do not do this as standard. 

Many physicians with good knowledge and training miss acute aortic dissection due to a lack of experience. It is not a common diagnosis, and many will go for a significant period of their careers without encountering it. Although building experience takes time and a degree of fortune, building a good foundation during medical education is highly important. It is clear from our data that better education is required, a finding which is corroborated by wider studies undertaken by the Aortic Dissection Collaborative [12]. They have suggested that future education should follow the examples of other medical specialities such as stroke, diabetes, and cancer. These specialities use patient information, social media, and other resources such as YouTube (Google LLC, Mountain View, California, United States) to disseminate information to students, clinicians, and patients alike, although the quality and consistency of this content with regards to vascular surgery is poor at present [13]. 

There are likely to be multiple impediments to quality education surrounding aortic dissection. There is some debate as to the most effective way of screening, preventing, diagnosing, and also treating a dissection [14]. Many myths exist regarding the prognosis and prevalence of aortic dissection, namely that it is very rare and almost always fatal, which contributes to a lack of awareness and apathy. More quality research, utilizing standardized evaluation tools, is needed to improve the standard of education regarding aortic dissection. 

Limitations

We appreciate that this study had a relatively small sample size and that the design lacked a degree of flexibility and dynamism, in that students weren’t being given red flags as they discovered them, more akin to real life. It would be more revealing to run the scenario as a practical station and see how students worked through the problem in more detail. Additionally, although we wanted students with the appropriate level of training, our exclusion criteria may not be foolproof as different medical schools provide different curriculums. We hope that this data will allow us to conduct more research into the recognition of acute aortic dissection and the possible development of a red flag system, which will reduce the number of missed cases.

## Conclusions

For the most part, chest pain is well taught across UK medical schools, with the majority of students considering myocardial infarction and pulmonary embolism as significant differential diagnoses. Students are asking appropriate questions and suggesting appropriate investigations.

However, we believe the way aortic dissection is currently taught across medical schools does not place enough emphasis on the severity of the condition and that further education is required to ensure it forms part of their diagnostic workup and is considered as a differential to avoid a missed diagnosis. The study also suggests that students are only considering the most likely diagnosis and are not looking to rule out conditions with higher mortality. Multiple impediments to quality education surrounding aortic dissection exist; however, more research needs to be carried out to confirm this and to elucidate the most effective method of combating the problem.

## References

[1] Walters K, Rait G, Hardoon S, Kalaitzaki E, Petersen I, Nazareth I (2014). Socio-demographic variation in chest pain incidence and subsequent coronary heart disease in primary care in the United Kingdom. Eur J Prev Cardiol.

[2] Croskerry P (2003). The importance of cognitive errors in diagnosis and strategies to minimize them. Acad Med.

[3] Tsai TT, Trimarchi S, Nienaber CA (2009). Acute aortic dissection: perspectives from the International Registry of Acute Aortic Dissection (IRAD). Eur J Vasc Endovasc Surg.

[4] Howard DP, Banerjee A, Fairhead JF, Perkins J, Silver LE, Rothwell PM (2013). Population-based study of incidence and outcome of acute aortic dissection and premorbid risk factor control: 10-year results from the Oxford Vascular Study. Circulation.

[5] Tsai TT, Nienaber CA, Eagle KA (2005). Acute aortic syndromes. Circulation.

[6] Gudbjartsson T, Ahlsson A, Geirsson A (2020). Acute type A aortic dissection - a review. Scand Cardiovasc J.

[7] Black JH, Manning WJ (2021). Clinical features and diagnosis of acute aortic dissection. UpToDate.

[8] Atkins MD Jr, Black JH 3rd, Cambria RP (2006). Aortic dissection: perspectives in the era of stent-graft repair. J Vasc Surg.

[9] Evangelista A, Maldonado G, Gruosso D, Teixido G, Rodríguez-Palomares J, Eagle K (2016). Insights from the International Registry of Acute Aortic Dissection. Glob Cardiol Sci Pract.

[10] (1997). World Medical Association declaration of Helsinki. Recommendations guiding physicians in biomedical research involving human subjects. JAMA.

[11] Hiratzka LF, Bakris GL, Beckman JA (2010). 2010 ACCF/AHA/AATS/ACR/ASA/SCA/SCAI/SIR/STS/SVM guidelines for the diagnosis and management of patients with thoracic aortic disease: a report of the American College of Cardiology Foundation/American Heart Association task force on practice guidelines, American Association for Thoracic Surgery, American College of Radiology, American Stroke Association, Society of Cardiovascular Anesthesiologists, Society for Cardiovascular Angiography and Interventions, Society of Interventional Radiology, Society of Thoracic Surgeons, and Society for Vascular Medicine. Circulation.

[12] Talutis SD, Watson J, Goldsborough E 3rd, Masciale E, Woo K (2022). Stakeholder perspectives on education in aortic dissection. Semin Vasc Surg.

[13] Phair J, Dalmia V, Sanon O (2021). The current state of vascular surgery presence and educational content in Google and YouTube internet search results. J Vasc Surg.

[14] Sayed A, Munir M, Bahbah EI (2021). Aortic dissection: a review of the pathophysiology, management and prospective advances. Curr Cardiol Rev.

